# Naltrexone and Bupropion Combination Treatment for Smoking Cessation and Weight Loss in Patients With Schizophrenia

**DOI:** 10.3389/fphar.2018.00181

**Published:** 2018-03-05

**Authors:** Xuechan Lyu, Jiang Du, Guilai Zhan, Yujie Wu, Hang Su, Youwei Zhu, Fredrik Jarskog, Min Zhao, Xiaoduo Fan

**Affiliations:** ^1^Shanghai Mental Health Center, Shanghai Jiao Tong University School of Medicine, Shanghai, China; ^2^Shanghai Xuhui District Mental Health Center, Shanghai, China; ^3^North Carolina Psychiatric Research Center, Department of Psychiatry, University of North Carolina at Chapel Hill, Chapel Hill, NC, United States; ^4^Shanghai Key Laboratory of Psychotic Disorders, Shanghai, China; ^5^Psychotic Disorders Program, UMass Memorial Medical Center, University of Massachusetts Medical School, Worcester, MA, United States

**Keywords:** Schizophrenia, smoking cessation, weight loss, naltrexone, bupropion

## Abstract

**Objective:** The rates of obesity and cigarette smoking are much higher in patients with schizophrenia compared to the general population. This study was to examine whether naltrexone and bupropion combination treatment can help weight loss and smoking cessation in patients with schizophrenia.

**Methods:** Obese male schizophrenia patients with current cigarette smoking were randomized to receive adjunctive naltrexone (25 mg/day) and bupropion (300 mg/day) combination or placebo for 24 weeks. Twenty-two patients were enrolled in the study, and 21 patients completed the study (11 in the treatment group, and 10 in the placebo group). Body weight, body mass index (BMI), fasting lipids, smoking urge, expired carbon monoxide (CO) level and cigarettes smoked per week were measured at baseline and week 24.

**Results:** There was no significant difference between two groups in changes in weight, BMI, fasting lipids, or cigarette smoking measures (*p*'s > 0.05)

**Conclusion:** Naltrexone and bupropion combination treatment didn't show weight loss or smoking cessation effect in patients with schizophrenia in this pilot study.Implications for future studies were discussed.

ClinicalTrials.gov identifier: NCT02736474.

## Introduction

Schizophrenia is a devastating neuropsychiatric disease with a prevalence of 1% worldwide. The rates of obesity are two times higher, and the rates of cigarette smoking are three to five times higher in this patient population compared to the general population, (Marder et al., 2004; Morisano et al., 2009). In a recent study of 896 Chinese patients with schizophrenia, 54% of them were found to be overweight or obese (Guo et al., 2013). The high rate of cigarette smoking was consistently reported in patients with schizophrenia across different cultures and countries (De Leon and Diaz, 2005). The prevalence of cigarette smoking in male schizophrenia patients in China is in the range of 54–79% but the prevalence in female schizophrenia patients is remarkably lower at 4–7% (Hou et al., 2011; Zhang et al., 2012). Notably, a common adverse effect of smoking cessation is weight gain (Yang et al., 2013). The average weight gain 6 months after the quit date is between 2.3 and 4.5 kg (Farley et al., 2009), which at least partially contributes to the difficulty in quitting smoking, especially in those who are already obese. Both obesity and cigarette smoking are significant risk factors for cardiovascular disease and premature death (Hruby et al., 2016; Cather et al., 2017). The mortality rate of schizophrenia patients is approximately twice that of the general population (Brown et al., 2000; Osby et al., 2000). Given the medical and economic burden of obesity and cigarette smoking in this patient population, establishing effective treatments targeting these two problems would have important public health implication.

Bupropion is an approved medication for depression and smoking cessation treatment. Bupropion can enhance subcortical dopamine and norepinephrine effects and mitigate the effects of nicotine-evoked dopamine transmission, therefore reduce nicotine reward and withdrawal experiences (Mansvelder et al., 2009). Studies have shown that bupropion is associated with a reduction in smoking related cue-induced activation of limbic and prefrontal brain regions (Culbertson et al., 2011). Clinically, bupropion is commonly used in individuals with schizophrenia for comorbid depression and/or smoking cessation. Recent studies have demonstrated that bupropion increases the rates of smoking abstinence and is well-tolerated in smokers with schizophrenia (Tsoi et al., 2010).

The mechanisms underlying weight gain post cessation in smokers are likely related to the results of heightened food reward (Lerman et al., 2004) and increased intake of palatable food with high sugar and fat contents (Hall et al., 1989). A competitive opioid antagonist such as naltrexone might affect opioidergic pathways and their connectivity to the midbrain reward circuit involved in motivational and hedonic aspects of feeding behaviors (Berridge et al., 2010). Recent studies suggested that, of the pharmacological treatments studied thus far to reduce weight gain after smoking cessation, the most promising results have been obtained with naltrexone (King et al., 2012, 2013).

Research on central nervous system pathways that regulate food intake and body weight has identified two key systems: the hypothalamic melanocortin system, which integrates input related to energy balance and produces anorexigenic signaling, and the mesolimbic reward system, which modulates reward value and goal-oriented behavior. Bupropion stimulates hypothalamic proopiomelanocortin (POMC) neurons while naltrexone can prevent inhibition of POMC neurons by blocking the action of β-endorphin (Billes et al., 2014). The combined treatment with sustained-release naltrexone (32 mg/day) and bupropion (360 mg/day) (NB) has proven to be effective in reducing body weight and improving cardiometabolic risks in obese individuals with or without diabetes (Greenway et al., 2010; Wadden et al., 2011; Hollander et al., 2013). The naltrexone and bupropion combination was used in an open label 24-week trial in overweight or obese nicotine-dependent subjects; the results showed significantly decreased nicotine use and craving without significant weight gain (Wilcox et al., 2010).

The combination of sustained-release naltrexone and bupropion (NB) (Contrave, Orexigen, CA) has received FDA-approval for weight loss in people who are overweight or obese (Greenway et al., 2010; Wadden et al., 2011). The purpose of the present 24-week, randomized, double blind, placebo controlled pilot trial was to examine the feasibility, safety, and initial benefit of using naltrexone and bupropion combination in treating obesity and smoking cessation in patients with schizophrenia.

## Methods

### Study participants

This study was approved by the ethics committee of Shanghai Mental Health Center. Each participant provided written informed consent. Male patients with schizophrenia were recruited from the inpatient unit at Xuhui Mental Health Center located in Shanghai, China. Each patient had 7 smoking breaks throughout the day from 6:15 a.m. to 7:30 p.m. During these 15-min breaks, patients were allowed to smoke cigarettes freely.

The inclusion criteria were: (1) diagnosis of schizophrenia by the International Classification of Diseases 10th Revision (ICD-10); (2) age between 18 and 65 years old; (3) on stable antipsychotic medication treatment for at least 1 month; (4) BMI > 28 kg/m^2^ according to BMI criterion for obesity in the Chinese population (Wu, 2006), or BMI > 27 kg/m^2^ in the presence of dyslipidemia, or male with waist circumference over 90 cm; (5) smoking at least 10 cigarettes daily for 1 year or longer; (6) desire to lose weight and quit smoking.

The exclusion criteria were: (1) eating disorders; (2) currently taking weight loss medications; (3) substance use (except caffeine or nicotine); (4) clinically significant liver disease; (5) clinically significant thyroid dysfunction; (6) history of epilepsy; (7) history of unstable heart diseases or other unstable medical conditions.

A total number of 330 male patients with schizophrenia were approached and screened between July 2016 and March 2017. Among 28 patients who fit the inclusion and exclusion criteria, 22 of them were enrolled in the study. One patient in the placebo group dropped out of the study after week-12 because of loss of follow up after being discharged from the inpatient unit. Therefore 21 patients (11 in the treatment group, 10 in the control group) completed the 24-week trial and were included in the final analysis.

### Intervention

Following screening and confirmation that inclusion and exclusion criteria were met, participants were randomized to receive the combination treatment or placebo. Participants were started with naltrexone 15 mg and bupropion sustained release 150 mg daily in the first 2 weeks. Then naltrexone was increased to 15 mg in the morning, 10 mg in the afternoon, and bupropion increased to 150 mg twice per day as tolerated during the remaining weeks of the study. Dosing targets were determined based on the efficacy and side effects associated with various dosing strategies reported in previous studies (Anderson et al., 2002; Greenway et al., 2009), as well as the available supplies of naltrexone and bupropion in China. Subjects were maintained on the same antipsychotic medication at the same dosage when clinically appropriate during the study.

### Outcome measures

Medical and psychiatric histories were obtained, and physical exam was performed for each participant. Urine drug screen and 12-lead EKG were performed at baseline. The following measures were completed at baseline and week 24: (1) height, weight and waist circumference; (2) fasting lipids, fasting glucose, HbA1c, and insulin; (3) breath CO level; (4) smoking craving using the Visual Analogue Scale; (5) number of cigarettes smoked in the past week; (6) the Positive and Negative Syndrome Scale (PANSS), (7) the side effects questionnaire.

### Laboratory assays

Laboratory assays were performed by the Bio-Chemistry Lab of Shanghai Mental Health Center. Fasting plasma glucose was measured with a hexokinase reagent kit. Hemoglobin A1C (HbA1c) was measured by high performance liquid chromatography with an automated analyzer. Fasting total plasma cholesterol and triglyceride levels were measured enzymatically, and the HDL cholesterol fraction was measured after precipitation of low-density lipoprotein (LDL) and very-low-density lipoprotein (VLDL) with dextran sulfatemagnesium, LDL levels were determined by the direct LDL reagents. Fasting insulin was measured using immunochromatography.

### Statistical analysis

Data were analyzed using SPSS 24.0 (IBM Corp., Armonk, NY). For baseline between-group comparisons, independent student *t*-test was used for continuous variables and chi-square test for categorical variables. Analysis of covariance (ANCOVA) was used to examine the between-group differences in changes of outcome measures from baseline to week 24 controlling for baseline values and other potential confounding variables. A *p* < 0.05 (two-sided) was considered statistically significant. Because this was designed as an exploratory pilot study, adjustments were not made for multiple comparisons.

## Results

### Baseline characteristics

There were no significant differences between the two groups on age, illness time duration, smoking time duration, breath CO level, number of cigarettes smoked per week, smoking craving, body weight, BMI, waist circumference, HbA1c, fasting glucose, HDL, LDL and insulin, and the PANSS total and subscale scores (*p*'s > 0.05). But the treatment group had significantly higher levels of education and triglycerides than the placebo group (*p* = 0.049 and *p* = 0.050, respectively) (Table 1). In the treatment group, 5 patients were on clozapine, 7 on olanzapine, 8 on other antipsychotic agents (risperidone, amisulpride, quetiapine, and chlorpromazine); in the placebo group, 5 patients were on clozapine, 4 on olanzapine, 11 on other antipsychotic agents (risperidone, amisulpride, paliperidone, and chlorpromazine. There were no significant differences between the two groups in the numbers of patients on clozapine or olanzapine (*p*'s > 0.05).

**Table 1:** Baseline demographic and general clinical characteristics.

**Variable**	**Treatment group (*N* = 11) (Mean + *SD*)**	**Placebo group (*N* = 10) (Mean + *SD*)**	***P*-value**
Age (years)	54.3 ± 5.7	56.2 ± 4.8	0.418
Education (years)	11.0 ± 2.8	8.1 ± 3.5	0.049^*^
Illness time duration (years)	29.8 ± 4.6	27.0 ± 8.8	0.400
Smoking time duration (years)	28.6 ± 7.8	32.4 ± 4.9	0.211
Breath CO Level (ppm)	27.1 ± 23.2	23.8 ± 9.0	0.679
Number of cigarettes smoked per week	72.5 ± 5.7	77.0 ± 22.1	0.526
VAS smoking craving	6.3 ± 1.0	7.0 ± 1.4	0.202
Weight (kg)	74.7 ± 8.4	78.4 ± 10.6	0.380
BMI (kg/m^2^)	26.1 ± 2.9	27.6 ± 2.6	0.229
waist circumference (cm)	94.9 ± 4.5	98.8 ± 6.3	0.118
PANSS-Total	51.5 ± 17.4	55.9 ± 20.3	0.602
PANSS-Positive	10.0 ± 3.4	11.2 ± 6.1	0.579
PANSS-Negative	15.9 ± 5.4	19.9 ± 7.9	0.188
PANSS-General	24.3 ± 9.1	25.3 ± 11.1	0.819
Glucose (mmol/l)	5.5 ± 1.1	5.6 ± 2.3	0.903
HbA1c (%)	6.1 ± 0.6	6.4 ± 1.9	0.729
Triglyceride (mmol/l)	2.3 ± 1.1	1.4 ± 0.6	0.050^*^
HDL (mmol/l)	0.9 ± 0.2	0.8 ± 0.1	0.163
LDL (mmol/l)	2.8 ± 0.8	2.4 ± 0.9	0.234
Insulin (pmol/l)	106.8 ± 65.8	66.4 ± 20.2	0.095

*VAS, visual analogue scale; BMI, body mass index; PANSS, the positive and negative syndrome scale; HDL, high density lipoprotein; LDL, Low-density lipoprotein*.

### Outcome measures after 24-week treatment

After controlling for education level and baseline value, the ANCOVA analysis showed no significant differences between the two groups in week 24 changes for metabolic measures including body weight, BMI, waist circumference, fasting glucose, HbA1c, triglycerides, HDL, LDL, and insulin (*p*'s > 0.05) (Table 2). At week 24, none of participants had successfully stopped smoking. After controlling for education level and baseline value, the ANCOVA analysis found no significant differences between the two groups in week 24 changes for breath CO level, number of cigarettes smoked per week, and smoking craving.

**Table 2:** Comparison of change values from baseline to week 24 on outcome measures.

**Outcome measure**	**Change value (week 24 min baseline)**		
	**Treatment group**	**Placebo group**	***p***
	***N* = 11**	***N* = 10**	
	**(Mean + *SD*)**	**(Mean + *SD*)**	
Body weight (kg)	0.2 ± 5.3	−0.9 ± 3.1	0.779
BMI (kg/m^2^)	0.0 ± 1.9	−0.3 ± 1.1	0.314
Glucose	0.5 ± 1.5	1.0 ± 1.6	0.264
HbA1c	0.05 ± 0.6	0.4 ± 1.1	0.385
Triglycerides	−1.0 ± 0.9	−0.4 ± 0.6	0.591
HDL	0.0 ± 1.1	0.0 ± 0.1	0.657
LDL	−0.4 ± 0.7	−0.2 ± 0.7	0.900
Insulin	3.5 ± 74.4	11.0 ± 35.3	0.262
Breath CO level (ppm)	−5.3 ± 22.4	−7.2 ± 7.4	0.289
Cigarettes per week	−6.2 ± 16.3	−6.3 ± 23.0	0.621
Craving scores	−1.0 ± 2.9	−1.9 ± 3.1	0.481

*Group comparisons were performed using ANCOVA controlling for baseline value and education level*.

### Adverse reactions

During the 24-week study time period, five participants reported dry mouth and1 participant reported mild abdominal discomfort in the treatment group; 1 participant reported tachycardia in the placebo group. There were no serious adverse events reported during the study. No participants withdrew from the study because of side effects.

## Discussion

Obesity and tobacco dependence are two major clinical challenges in patients with schizophrenia. To date studies have generally focused on addressing one or the other but not both. Identifying effective strategies to address both problems simultaneously would have great clinical value and public health implication. The current pilot study was designed to test naltrexone and bupropion combination treatment to address obesity and tobacco dependence together in individuals with schizophrenia. To our knowledge, this was the first study to target two difficult-to-treat conditions simultaneously in this population. Our study suggests that naltrexone and bupropion combination is well-tolerated in patients with schizophrenia.

Our study failed to demonstrate benefit of naltrexone and bupropion combination treatment for weight loss or smoking cessation in patients with schizophrenia. Possible reasons may include the small sample size, dosing of study medication, peer pressure to engage in cigarette smoking in the inpatient environment, and the absence of a psychosocial intervention combined with the pharmacological intervention. Another possibility is that the mechanisms related to obesity and cigarette smoking in patients with schizophrenia may not be responsive to naltrexone and bupropion combination treatment. The high rates of tobacco dependence in individuals with schizophrenia are believed to result from a range of neurobiological vulnerability factors that likely act in concert (Wing et al., 2012). Two overarching theories have been proposed to explain the high rates of comorbid tobacco addiction in schizophrenia. The self-medication hypothesis proposes that people with schizophrenia smoke in an attempt to improve psychiatric symptoms (Winterer, 2010). The addiction vulnerability hypothesis proposes that common abnormalities in the brain make patients with schizophrenia more vulnerable to tobacco use (Wing et al., 2012). Previous studies have demonstrated that treatment with first- and second-generation antipsychotics contributes to weight gain and obesity (Allison and Casey, 2001). In our study, ongoing antipsychotic treatment may have counteracted the possible weight loss effect of naltrexone and bupropion combination treatment. Other possible limitations include: (1) Patients in the study were able to smoke only during smoking breaks rather than smoke freely; the smoking restriction might have affected the smoking cessation outcome measure. (2) Even though the desire to lose weight and quit smoking was required for study participation, the level of motivation, which is an important confounding factor, was not assessed quantitatively in the present study. (3) The study included male patients only due to the low prevalence of female smokers in China; it is unclear whether or not there is a gender difference in treatment response to naltrexone and bupropion combination.

Obesity and tobacco dependence are two major clinical challenges in patients with schizophrenia. To date studies have singly focused on addressing one or the other. Novel strategies to address both problems simultaneously would have high clinical value and considerable public health implication. Future studies with larger sample sizes and in combination with psychosocial interventions, such as motivational interviewing and/or nutritional counseling, are warranted to further explore potential benefits of naltrexone and bupropion combination treatment in treating obesity and cigarette smoking in patients with schizophrenia.

## Author contributions

FJ, XF, MZ: conceived and designed the experiments; XL, GZ, YW, HS, YZ: performed the experiments; XL: analyzed the data; XL, JD: contributed reagents, materials, analysis tools; XL, JD, XF: wrote the paper.

### Conflict of interest statement

The authors declare that the research was conducted in the absence of any commercial or financial relationships that could be construed as a potential conflict of interest.

## References

[B1] Allison D. B. Casey D. E. (2001). Antipsychotic-induced weight gain: a review of the literature. J. Clin. Psychiatry 62(Suppl. 7.), 22–31. 11346192

[B2] Anderson J. W. Greenway F. L. Fujioka K. Gadde K. M. McKenney J. O'Neil P. M. (2002). Bupropion SR enhances weight loss: a 48-week double-blind, placebo- controlled trial. Obes. Res. 10, 633–641. 10.1038/oby.2002.86 12105285

[B3] Berridge K. C. Ho C. Y. Richard J. M. Difeliceantonio A. G. (2010). The tempted brain eats: pleasure and desire circuits in obesity and eating disorders. Brain Res. 1350, 43–64. 10.1016/j.brainres.2010.04.003 20388498 PMC2913163

[B4] Billes S. K. Sinnayah P. Cowley M. A. (2014). Naltrexone/bupropion for obesity: an investigational combination pharmacotherapy for weight loss. Pharmacol. Res. 84, 1–11. 10.1016/j.phrs.2014.04.004 24754973

[B5] Brown S. Inskip H. Barraclough B. (2000). Causes of the excess mortality of schizophrenia. Br. J. Psychiatry 177, 212–217. 10.1192/bjp.177.3.212 11040880

[B6] Cather C. Pachas G. N. Cieslak K. M. Evins A. E. (2017). Achieving Smoking Cessation in Individuals with Schizophrenia: special Considerations. CNS Drugs 31, 471–481. 10.1007/s40263-017-0438-8 28550660 PMC5646360

[B7] Culbertson C. S. Bramen J. Cohen M. S. London E. D. Olmstead R. E. Gan J. J.. (2011). Effect of bupropion treatment on brain activation induced by cigarette-related cues in smokers. Arch. Gen. Psychiatry 68, 505–515. 10.1001/archgenpsychiatry.2010.193 21199957 PMC3214639

[B8] de Leon J. Diaz F. J. (2005). A meta-analysis of worldwide studies demonstrates an association between schizophrenia and tobacco smoking behaviors. Schizophr. Res. 76, 135–157. 10.1016/j.schres.2005.02.010 15949648

[B9] Farley A. C. Hajek P. Lycett D. Aveyard P. (2009). Interventions for preventing weight gain after smoking cessation. Cochrane Database Syst Rev. 1:CD006219. 10.1002/14651858.CD006219.pub3 22258966

[B10] Greenway F. L. Fujioka K. Plodkowski R. A. Mudaliar S. Guttadauria M. Erickson J.. (2010). Effect of naltrexone plus bupropion on weight loss in overweight and obese adults (COR-I): a multicentre, randomised, double-blind, placebo-controlled, phase 3 trial. Lancet 376, 595–605. 10.1016/S0140-6736(10)60888-4 20673995

[B11] Greenway F. L. Whitehouse M. J. Guttadauria M. Anderson J. W. Atkinson R. L. Fujioka K.. (2009). Rational design of a combination medication for the treatment of obesity. Obesity 17, 30–39. 10.1038/oby.2008.461 18997675

[B12] Guo X. Zhang Z. Wei Q. Lv H. Wu R. Zhao J. (2013). The relationship between obesity and neurocognitive function in Chinese patients with schizophrenia. BMC Psychiatry 13:109. 10.1186/1471-244X-13-109 23570390 PMC3627610

[B13] Hall S. M. McGee R. Tunstall C. Duffy J. Benowitz N. (1989). Changes in food intake and activity after quitting smoking. J. Consul. Clin. Psychol. 57, 81–86. 10.1037/0022-006X.57.1.81 2925977

[B14] Hollander P. Gupta A. K. Plodkowski R. Greenway F. Bays H. Burns C.. (2013). Effects of naltrexone sustained-release/bupropion sustained-release combination therapy on body weight and glycemic parameters in overweight and obese patients with type 2 diabetes. Diabetes Care 36, 4022–4029. 10.2337/dc13-0234 24144653 PMC3836105

[B15] Hou Y. Z. Xiang Y. T. Yan F. Ungvari G. S. Dickerson F. Chiu H. F.. (2011). Cigarette smoking in community-dwelling patients with schizophrenia in China. J. Psychiatr. Res. 45, 1551–1556. 10.1016/j.jpsychires.2011.07.011 21820671

[B16] Hruby A. Manson J. E. Qi L. Malik V. S. Rimm E. B. Sun Q.. (2016). Determinants and consequences of obesity. Am. J. Public Health 106, 1656–1662. 10.2105/AJPH.2016.303326 27459460 PMC4981805

[B17] King A. C. Cao D. O'Malley S. S. Kranzler H. R. Cai X. dewit H.. (2012). Effects of naltrexone on smoking cessation outcomes and weight gain in nicotine-dependent men and women. J. Clin. Psychopharmacol. 32, 630–636. 10.1097/JCP.0b013e3182676956 22926596 PMC4640209

[B18] King A. C. Cao D. Zhang L. O'Malley S. S. (2013). Naltrexone reduction of long-term smoking cessation weight gain in women but not men: a randomized controlled trial. Biol. Psychiatry. 73, 924–930. 10.1016/j.biopsych.2012.09.025 23177384 PMC3629005

[B19] Lerman C. Berrettini W. Pinto A. Patterson F. Crystal-mansour S. Wileyto E. P.. (2004). Changes in food reward following smoking cessation: a pharmacogenetic investigation. Psychopharmacology 174, 571–577. 10.1007/s00213-004-1823-9 15138759

[B20] Mansvelder H. D. Mertz M. Role L. W. (2009). Nicotinic modulation of synaptic transmission and plasticity in cortico-limbic circuits. Semin. Cell Dev. Biol. 20, 432–440. 10.1016/j.semcdb.2009.01.007 19560048 PMC2742626

[B21] Marder S. R. Essock S. M. Miller A. L. Buchanan R. W. Casey D. E. Davis J. M.. (2004). Physical health monitoring of patients with schizophrenia. Am. J. Psychiatry 161, 1334–1349. 10.1176/appi.ajp.161.8.1334 15285957

[B22] Morisano D. Bacher I. Audrain-McGovern J. George T. P. (2009). Mechanisms underlying the comorbidity of tobacco use in mental health and addictive disorders. Can. J. Psychiatry 54, 356–367. 10.1177/070674370905400603 19527556

[B23] Osby U. Correia N. Brandt L. Ekbom A. Sparén P. (2000). Mortality and causes of death in schizophrenia in Stockholm county, Sweden. Schizophr. Res. 45, 21–28. 10.1016/S0920-9964(99)00191-7 10978869

[B24] Tsoi D. T. Porwal M. Webster A. C. (2010). Efficacy and safety of bupropion for smoking cessation and reduction in schizophrenia: systematic review and meta-analysis. Br. J. Psychiatry 196, 346–353. 10.1192/bjp.bp.109.066019 20435957

[B25] Wadden T. A. Foreyt J. P. Foster G. D. Hill J. O. Klein S. O'Neil P. M.. (2011). Weight loss with naltrexone SR/bupropion SR combination therapy as an adjunct to behavior modification: the COR-BMOD trial. Obesity 19, 110–120. 10.1038/oby.2010.147 20559296 PMC4459776

[B26] Wilcox C. S. Oskooilar N. Erickson J. S. Billes S. K. Katz B. B. Tollefson G.. (2010). An open-label study of naltrexone and bupropion combination therapy for smoking cessation in overweight and obese subjects. Addict. Behav. 35, 229–234. 10.1016/j.addbeh.2009.10.017 19926400

[B27] Wing V. C. Wass C. E. Soh D. W. George T. P. (2012). A review of neurobiological vulnerability factors and treatment implications for comorbid tobacco dependence in schizophrenia. Ann. N. Y. Acad. Sci. 1248, 89–106. 10.1111/j.1749-6632.2011.06261.x 22129082

[B28] Winterer G. (2010). Why do patients with schizophrenia smoke? Curr. Opin. Psychiatry 23, 112–119. 10.1097/YCO.0b013e3283366643 20051860

[B29] Wu Y. (2006). Overweight and obesity in China. BMJ 333, 362–363. 10.1136/bmj.333.7564.362 16916811 PMC1550451

[B30] Yang M. Bhowmik D. Wang X. Abughosh S. (2013). Does combination pharmacological intervention for smoking cessation prevent post-cessation weight gain? a systemic review. Addict. Behav. 38, 1865–1875. 10.1016/j.addbeh.2012.11.007 23305808

[B31] Zhang X. Y. Liang J. C. Chen D. C. Xiu M. H. He J. Cheng W.. (2012). Cigarette smoking in male patients with chronic schizophrenia in a Chinese population: prevalence and relationship to clinical phenotypes. PLoS ONE 7:e30937. 10.1371/journal.pone.0030937 22347412 PMC3274516

